# Rabies vaccination induces a CD4^+^ T_EM_ and CD4^+^CD8^+^ T_EMRA_ T_H_1 phenotype in dogs

**DOI:** 10.1371/journal.pone.0323823

**Published:** 2025-05-12

**Authors:** Haeree P. Lang, Farah F. Almeer, Marc K. Jenkins, Steven G. Friedenberg

**Affiliations:** 1 Center for Immunology, University of Minnesota, Minneapolis, Minnesota, United States of America; 2 Department of Veterinary Clinical Sciences, College of Veterinary Medicine, University of Minnesota, St. Paul, Minnesota, United States of America; University of California Santa Barbara, UNITED STATES OF AMERICA

## Abstract

The canine rabies vaccine consists of the whole killed rabies virus and an alum adjuvant. While it is known to provide immunological protection in dogs, its effects on cell-mediated responses remain largely uncharacterized. Here, we analyzed blood and spleen samples from vaccinated dogs to understand adaptive immune responses *ex vivo* following restimulation with rabies vaccine antigens. Our results showed that recombinant rabies virus glycoprotein (RABV-G) elicited higher antibody titers and IFNγ production compared to recombinant rabies virus nucleoprotein (RABV-N). CD4^+^ and CD4^+^CD8^+^ double-positive (DP) T cells proliferate robustly after five days of RABV-G stimulation, which was inhibited by an anti-canine MHC class II blocking antibody. Both RABV-G-specific CD4^+^ and DP T cells demonstrated a polarized T_H_1 phenotype, with minor subsets showing T_H_1/T_H_17 hybrid and pathogenic T_H_1/T_H_17 hybrid cell features. CD4^+^ T cells were primarily effector memory T cells (T_EM_), while DP T cells exhibited a terminally differentiated effector memory phenotype that re-expressed CD45RA (T_EMRA_). Both RABV-G-specific CD4^+^ and DP T cells were detectable up to 1,024 days post-vaccination in spleen samples and their proliferative capacities were unaffected by age. Our results provide the first characterization of canine RABV-G-specific T cell phenotypes in the spleen and blood following rabies vaccination.

## Introduction

Millions of dogs are vaccinated annually to provide protection from potentially fatal diseases [1]. While neutralizing antibody titers in dogs are often seen as a primary correlate of protection [2], vaccines also induce robust T cell responses [3,4]. The protective role of vaccine-induced T cells is poorly understood in dogs, aside from their function in promoting antibody production [5,6]. Studies in humans and mice have demonstrated that antigen-specific CD4^+^ T cells are crucial for vaccine-mediated protection and maintenance of long-term immunological memory [7]. Depleting CD4^+^ T cells in mice during the immunization phase results in a loss of protection against viral challenge [8–10]. Similarly, people with severe idiopathic CD4 lymphopenia or uncontrolled HIV have blunted vaccine-induced immune responses [11,12]. However, the role of T cells in vaccine-induced immunity can be challenging to study due to variations in the route of administration, type of vaccine and adjuvant, and the number and timing of doses within the vaccination series.

Dogs are routinely vaccinated every 1–3 years with long-standing formulations, and therefore may serve as a reliable animal model for exploring T cell behavior following vaccination [13]. The most commonly administered vaccine in dogs is the rabies vaccine, which contains the whole killed virus and alum adjuvant given subcutaneously [14]. This formulation is specific to companion animals. Humans receive cell culture-derived, non-adjuvanted rabies vaccines intramuscularly that have been shown to elicit type 1 helper (T_H_1) responses and neutralizing antibodies against the rabies virus glycoprotein and nucleoprotein [8,9,15–19]. Although the canine rabies vaccine also induces neutralizing antibodies against these proteins, studies in dogs have primarily documented bulk T cell responses *ex vivo* without identifying specific subsets. Additionally, most studies have focused on experimental vaccines rather than those used in clinical practice [20–26].

To address this knowledge gap, we characterized memory and lineage T cell subsets in peripheral blood mononuclear cells (PBMCs) from recently vaccinated dogs, as well as in spleen samples from dogs vaccinated months to years before splenocyte collection. For this purpose, we developed a comprehensive flow cytometry panel to define canine T cells by lineage-defining transcription factors, cytokines, and memory markers. We show that rabies vaccine-specific T cells proliferate readily *in vitro* using a dye dilution assay, and that they are predominantly CD4^+^CD8^+^ double-positive (DP) T_H_1 terminally differentiated effector memory T cells that re-expressed CD45RA (T_EMRA_) and CD4^+^ T_H_1 effector memory (T_EM_) T cells. These findings provide insights into how the rabies vaccine elicits potent long-lasting T cell responses in CD4^+^ T cells and highlights the unexpected role of DP T cells in rabies vaccine-induced immunity.

## Materials and methods

### Animals

Client-owned dogs scheduled for rabies booster vaccination at the University of Minnesota College of Veterinary Medicine (UMN CVM) Primary Care Service were prospectively recruited. Dogs were eligible if they weighed at least 20 kg, were at least 3 years of age, and had received at least one prior rabies vaccine. Dogs were excluded if deemed unwell based on a physical examination by the attending veterinarian. On the day of vaccination, 8 mL of blood was collected into CPT Mononuclear Cell Preparation Tubes (sodium citrate, 362761, BD) and processed immediately or stored at 4°C for up to 24 hours. Enrolled dogs returned 7–14 days later for a second blood draw (2 mL/kg, up to 50 mL), timed to capture peak antigen-specific CD4^+^ T cell expansion post-vaccination rather than peak antibody production [27,28]. These samples were collected into syringes prefilled with lithium heparin anticoagulant. Blood collection was performed with minimal restraint and animals were returned to their owners following sampling. All procedures were approved by the UMN Institutional Animal Use and Care Committee (protocol #2107-39275A). Blood collection was minimally invasive, involving standard venipuncture without anesthesia, analgesia, or euthanasia, and efforts were made to minimize discomfort.

Splenic tissue was collected from two separate cohorts of dogs. Dogs from the first cohort were undergoing medically indicated splenectomies for isolated, encapsulated masses; grossly healthy tissue distant from the mass was obtained intraoperatively. These dogs received standard-of-care anesthesia and post-operative analgesia; they were cared for in the intensive care unit after surgery and returned to their owners at the discretion of the attending veterinarian. Splenic tissue from the second cohort of dogs were obtained post-mortem immediately after euthanasia from dogs whose bodies were donated for research. Details regarding the procurement source of each spleen are available in S1 Table. Euthanasia decisions were made independently by the dog’s owner based on medical considerations unrelated to this study. Details regarding enrolled dogs are provided in S2 Table (PBMCs) and S1 Table (splenocytes).

### Sample processing

PBMCs from both Day 0 and Day 7–14 time points were isolated in 50 mL Leucosep^®^ tubes following manufacturer's protocols (Z642843, Greiner). 15 mL of Ficoll-Paque Plus™ (17144003, Cytiva) was added to the Leucosep^®^ tube and centrifuged at 1000 x g for 30 seconds. Whole blood was diluted 1:1 with PBS. The sample was centrifuged with the brake disabled. Plasma was carefully removed and kept frozen at -20ºC until use in IgG titer ELISA assays. The enriched cell fraction was removed and washed twice in cold PBS.

For splenic samples, approximately 3–6 2 x 2 x 0.5 cm^3^ pieces of tissue were minced in a cell culture dish containing cold RPMI 1640 media and passed through a 70 μM nylon cell strainer (08-771-2, ThermoFisher Scientific). Single cell suspensions were resuspended in RPMI media and centrifuged. The splenocyte pellet was treated with ACK lysis buffer (A1049201, ThermoFisher Scientific) for 5 min. The lysis reaction was quenched with media and centrifuged again. ACK lysis buffer treatment was repeated once more.

Cell viability and cell concentration for all samples were determined with Trypan Blue stain on a hemocytometer. Cells were cryopreserved in liquid nitrogen with freezing media containing 90% fetal bovine serum (FBS, Omega Scientific) and 10% dimethyl sulfoxide (DMSO, Sigma Aldrich).

### Rabies IgG antibody ELISA

Plasma-derived antibody binding against RABV-G and RABV-N was assessed by enzyme-linked immunosorbent assay (ELISA). 96-well plates were coated with 2 μg/mL of commercially available recombinant RABV-G (ab225663, Abcam) or RABV-N (LS-G21410-20, LSBio). Recombinant RABV-G was derived from the Pasteur strain, the same strain used in the canine rabies vaccine, whereas recombinant RABV-N (LSBio) was derived from the Pitman-Moore 1503 strain which shares 98% identity with the Pasteur strain (S7 Fig) [29]. Duplicated 6-point serial dilutions were performed on thawed plasma samples using a blocking buffer consisting of 1% bovine serum albumin (BSA). Detection was performed with a goat anti-canine IgG HRP-conjugated secondary antibody (A18763, Invitrogen). Plates were incubated with TMB Super Sensitive One Component HRP Microwell Substrate (TMBS-1000–01, Surmodics) to induce a chromogenic reaction and quenched with a 450 nm Liquid Stop Solution (LSTP-1000–01, Surmodics). Absorbance was read at 450 nm using an ELISA plate reader. In all assays, a plasma sample from an unvaccinated puppy was used as a negative control. For IgG titers, the half-maximal effective concentration (EC_50_) was calculated for each sample based on the entire dilution series. Differences between Day 0 and Day 7–14 titers against time RABV-G and RABV-N were compared using a paired two-tailed Wilcoxon test. For all statistical tests, here and subsequently, *p *< 0.05 was considered significant.

### IFNγ ELISpot assay

A canine IFNγ ELISpot Development Module (SEL781, R&D Systems) was used per the manufacturer’s protocol with slight modifications. Briefly, an 8-strip Immobilon-P^®^ hydrophobic polyvinylidene fluoride (PVDF) membrane microplate (M8IPS4510, Millipore) was pre-wet with 35% ethanol, washed with PBS, coated with anti-canine IFNγ capture antibody (840804, R&D Systems), and incubated at 4°C overnight. After washing and blocking with 1% BSA, 5% sucrose in PBS for two hours, 2.5–5 x 10^5^ PBMCs were plated per well. DMSO (0.1% total concentration) was used as a negative control, and RABV-G (ab225663, Abcam) or RABV-N (LS-G21410-20, LSBio) were added at a 2 μg/mL. Cells incubated undisturbed for 18–24 hours at 37°C and 5% CO_2_. Following three washes, canine IFNγ detection antibody (840805, R&D Systems) was added and incubated at 4°C overnight. Plates were washed and developed using the ELISpot Blue Color Module (SEL002, R&D Systems). Spots were counted using a CTL ImmunoSpot^®^ Analyzer, and IFNγ spot numbers between RABV-G- and RABV-N-stimulated PBMCs were compared using a paired, two-tailed Wilcoxon test.

### Cell proliferation assay

Cryopreserved PBMCs or splenocytes were thawed in a 37°C water bath and washed with warmed complete RPMI 1640 (cRPMI) media containing 25 mM HEPES buffer, 300 μg/mL L-glutamine, 10% HI-FBS, 1% sodium pyruvate, 1% non-essential amino acids, 100 U/mL penicillin, 100 μg/mL streptomycin, and 55 μM β-mercaptoethanol (B-ME). After centrifugation, cells were resuspended in PBS containing 1 μM of CellTrace Far Red (CTFR) dye (C34572, Invitrogen) and incubated at 37°C for 20 minutes. Cells were washed with 5 times the original staining volume with 2% FBS in PBS and incubated for 5 minutes at 37°C before being centrifuged. Cells were resuspended in warmed cRPMI and rested for 10 minutes prior to stimulation of 2–5 x 10^6^ cells/mL with either *Staphylococcus* enterotoxin B (SEB) at 2 μg/mL as a positive control, RABV-G (ab225663, Abcam) at 2 μg/mL, or a 0.1% DMSO negative control, and placed in a 37°C 5% CO_2_ incubator for five days in cRPMI. For MHC class II (MHCII) blocking experiments, samples were stained with CTFR dye and incubated with 2 μg/mL of RABV-G and either 10 μg/mL of a rat anti-canine MHCII monomorphic antibody (YKIX334.2, unconjugated, BioRad) or 10 μg/mL of a mouse anti-rat IgG2a, κ isotype control (R35-95, unconjugated, BD).

Paired, two-tailed Wilcoxon tests were performed to compare the % of proliferated (CTFR^LO^) cells for the following experiments: mock-stimulated vs. RABV-G-stimulated within CD4^+^, DP, or CD8^+^ T cell subsets, isotype control vs. MHC class II-treated within mock- and RABV-G-stimulated groups, and %CTFR^HI^ vs. %CTFR^LO^ across T_N_, T_CM_, T_EM_, T_EMRA_, T_H_1, T_H_1/T_H_17, pathogenic T_H_1/T_H_17, and Treg subsets. Unpaired, two-tailed Mann-Whitney U tests were used to measure differences in % proliferated (CTFR^LO^) cells between PBMCs and splenocytes across all T cell subsets evaluated. A two-way ANOVA was used to compare % proliferated cells (CTFR^LO^) between mock- and RABV-G-stimulated CD4^+^, DP, and CD8^+^ T cells across six time points, as well as IFNγ and IL-17A MFI across the same time points. Linear regression models were applied to blood and spleen samples separately to assess the relationship between % proliferated (CTFR^LO^) cells and age (in years) or days since last vaccination. Raw proliferation data are provided in S4 Table.

### Flow cytometry

After five days of stimulation, cells were harvested and stained with canine Fc block (14-9162-42, ThermoFisher Scientific) for 10 minutes prior to the addition of CD45RA antibody (CA4.1D3, unconjugated, BioRad) for 15 minutes. Cells were then washed with sorter buffer, centrifuged and stained at 4°C for 20 minutes with an anti-mouse IgG1 (M1-14D12, Super Bright 780, Invitrogen) as a secondary antibody to detect CD45RA^+^ cells, along with other antibodies directed against CD62L (FMC46, StarBright UltraViolet 605, BioRad), CD4 (YKIX302.9, PE-Cy7, BioRad), CD8 (YCATE55.9, Pacific Blue, BioRad), CD11b (M1/70, FITC, BioLegend), CD21 (CA2.1D6, Alexa Fluor 488, BioRad), CD14 (TÜK4, FITC, BioRad), and with Zombie Green Fixable Viability Dye (BioLegend). Cells were then fixed for 1 hour and permeabilized with the Foxp3/Transcription Factor Staining Buffer Set (eBioscience) and stained overnight at 4°C with antibodies specific for T-bet (eBio4B10, PE-Cy5, Invitrogen), IFNγ (CC302, PE, Invitrogen), CD3 (CA17.2A12, Alexa Fluor 700, BioRad), IL-17A (eBio64DEC17, BUV737, Invitrogen) and FOXP3 (FJK-16s, PerCP-eFluor 710, Invitrogen). Flow cytometry was performed on a Cytek Aurora spectral flow cytometer (5L) and analyzed with FlowJo software (v 10.10.0).

## Results

### The rabies vaccine induces predominantly RABV-G specific antibodies

**Fig 1:**
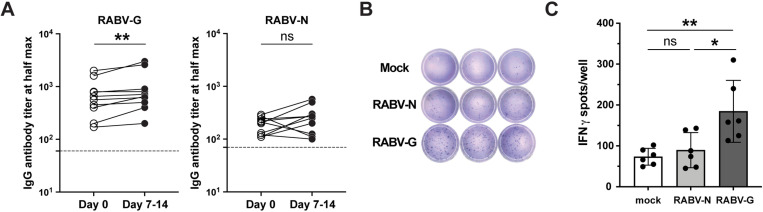
RABV-G generates a higher antibody titer post-vaccination than RABV-N. (A) Total IgG titers against RABV-G and RABV-N on the day of a rabies vaccine booster (Day 0) or 7-14 days after vaccine administration. The dashed line was determined by the antibody titer from an unvaccinated puppy (Dog 11, S3 Table) and was used as a normalization control for each experiment (*n *= 10, paired two-tailed Wilcoxon test; ns, not significant, **p < 0.01; two independent experiments). (B) Representative IFNγ ELISpot images of mock-, RABV-N-, and RABV-G- stimulated PBMCs performed in triplicate. (C) Quantification of the data represented in (B) (*n* = 6; one-way ANOVA and post hoc Tukey’s test; ns, not significant, *p < 0.05, **p < 0.01; three independent experiments).

The rabies virus glycoprotein (RABV-G) is recognized across species as the immunodominant protein in rabies vaccines, responsible for inducing potent virus-neutralizing antibodies [8,9,18,30–32]. However, studies have shown that antibodies against the rabies virus nucleoprotein (RABV-N) are also produced after vaccination [26,33,34]. To identify the immunodominant protein in our canine cohort, we measured antibody titers against recombinant RABV-G and RABV-N by ELISA at two time points: Day 0 (the day they received the rabies vaccine) and 7–14 days post-vaccination. In 10 dogs, RABV-N titers were consistently lower than RABV-G at both time points, and the vaccine booster did not significantly elevate RABV-N titers within the two-week timeframe (**Fig 1A** and S3 Table). Although all dogs showed increased RABV-G titers at Days 7–14, the level was lower than anticipated. This may be due to high baseline titers maintained by vaccinations every 1–3 years, likely neutralizing the vaccine quickly and limiting a robust recall response by memory B cells. Additionally, the peak of antibody production likely occurs later than 7–14 days post-boost, with the highest levels expected around Day 21–28 [35,36]. Nonetheless, these data identify RABV-G as the immunodominant protein, eliciting a stronger antibody response than RABV-N.

To evaluate cell-mediated responses, recombinant RABV-G and RABV-N were used to stimulate peripheral blood mononuclear cells (PBMCs) collected 7–14 days after vaccination. IFNγ production was measured 18–24 hours after *in vitro* stimulation by ELISpot in *n *= 5 dogs (**Fig 1B**). PBMCs produced significantly more IFNγ when stimulated with RABV-G compared to RABV-N (**Fig 1C**). These results provided additional evidence that RABV-G is the dominant protein driving immune responses following rabies vaccination.

### CD4^+^ and CD4^+^CD8^+^ double-positive T cells proliferate in response to RABV-G-specific peptide:MHCII interactions

To investigate the *in vitro* expansion of canine T cells, we optimized cell culture conditions for effective T cell proliferation. Our findings indicated that canine lymphocyte proliferation required β-mercaptoethanol (B-ME), a reducing agent that prevents disulfide bond formation and reduces oxidative stress. Without B-ME, dilution of CellTrace Far Red (CTFR) proliferation dye was severely diminished even when T cells were stimulated with Concanavalin A, phorbol 12-myristate 13-acetate (PMA) and ionomycin, or *Staphylococcus* enterotoxin B (SEB), which crosslinks T cell receptors and MHC class II molecules in a peptide-independent manner (S1 Fig).

**Fig 2:**
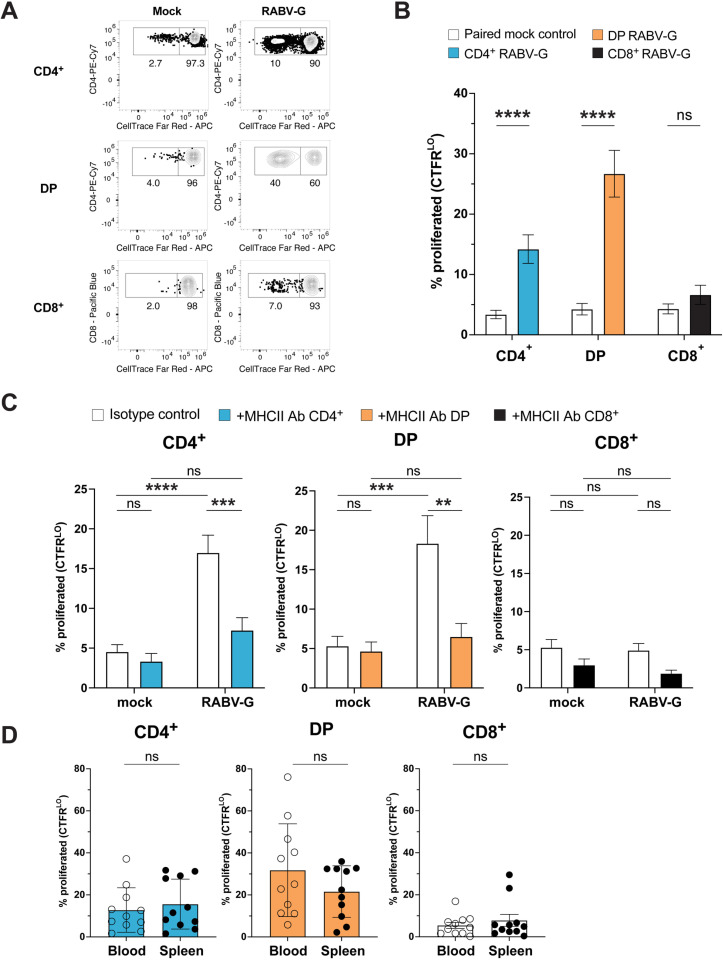
RABV-G stimulation induces the proliferation of CD4^+ ^and DP T cells. (A) Representative contour plots of proliferating (CTFR^LO^) versus non-proliferating (CTFR^HI^) T cell subsets in mock- and RABV-G-treated groups. (B) Quantification of (A) (mean ± SEM; *n *= 22; paired two-tailed Wilcoxon test; ns, not significant, ****p < 0.0001; 4 independent experiments). (C) Percentage of CTFR^LO^ T cell subsets treated with an isotype control or MHC class II antibody (mean ± SEM; *n* = 17; two-way ANOVA with multiple comparisons; ns, not significant, **p < 0.01, ***p < 0.001, ****p < 0.0001; 5 independent experiments). (D) Analysis by tissue type (blood vs. spleen) based on data from (B) (mean ± SEM; *n* = 22; two-tailed Mann-Whitney-U tests; ns, not significant; 4 independent experiments).

Once cell culture media conditions were optimized, we investigated which T cells were responding to RABV-G stimulation. We stained splenocytes or PBMCs with CTFR proliferation dye, stimulated them with RABV-G or a mock control for five days, and then assessed proliferating cells (CTFR^LO^) compared to non-proliferating cells (CTFR^HI^) by flow cytometry (S2 Fig) [37–39]. Although the overall percentages of CD4^+^, CD8^+^, and DP T cells did not increase following RABV-G-stimulation (S3 Fig), CD4^+^ and DP T cells proliferated significantly more than CD8^+^ T cells in response to RABV-G, as indicated by the higher percentage of CTFR^LO^ compared to CTFR^HI^ cells (**Figs 2A** and **2B**). The poor stimulation of CD8^+^ T cells was likely related to the fact that exogenously-added RABV-G would not be expected to enter the endogenous MHC class I antigen presentation pathway.

The RABV-G-stimulated proliferation of CD4^+^ and DP T cells was dependent on peptide:MHC class II interactions. Treatment with an anti-canine MHC class II blocking antibody significantly reduced the percentages of CTFR^LO^ CD4^+^ and DP T cells compared to an isotype control (**Fig 2C**). However, the MHC class II antibody did not reduce the percentages of mock- or RABV-G-stimulated CD8^+^ T cells, suggesting their minimal role in the response to RABV-G. Notably, there were no differences in CTFR^LO^ frequencies between blood- and spleen-derived CD4^+^, DP, and CTFR^LO^ CD8^+^ T cell subsets compared to their CTFR^HI^ counterparts, despite the spleen serving as a major reservoir of memory T cells (**Fig 2D**). Together, these results show that MHC class II-restricted DP and CD4^+^ T cells are the main responders to RABV-G stimulation *in vitro*.

### RABV-G-stimulated DP and CD4^+^ T cells surpass CD8^+^ cells in proliferation and cytokine production

**Fig 3:**
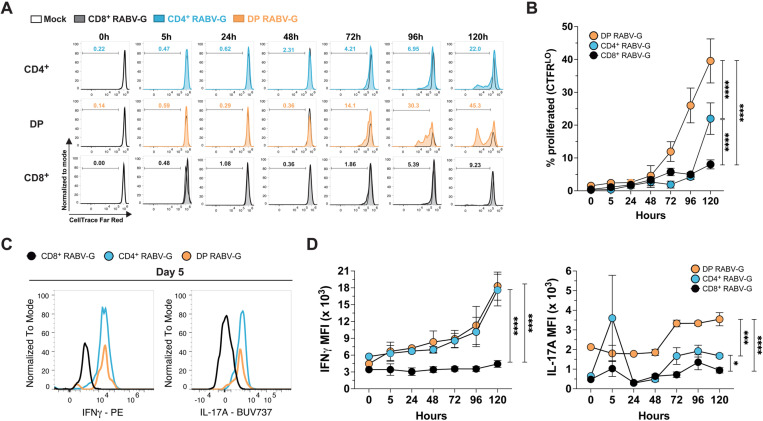
RABV-G-induced responses were apparent after 72 hours of stimulation. (A) Representative histogram plots compare the mock control (black outline) to RABV-G-stimulated T cell subsets: CD4^+^ (top row, blue), DP (middle row, orange), and CD8^+^ (bottom row, black), with % proliferated (CTFR^LO^) shown within the plot. (B) Quantification of (A) (mean ± SEM; *n *= 6/experiment; two-way ANOVA with multiple comparisons ****p < 0.0001; 6 independent experiments). Data was normalized to controls. (C) Representative histograms of IFNγ (left) and IL-17A (right) expression by CD8^+^, CD4^+^, or DP T cells stimulated with RABV-G after 5 days. (D) Mean fluorescence intensity (MFI) of IFNγ (left) and IL-17A (right) for CD4^+^ T cells, DP T cells, and CD8^+^ T cells (mean ± SEM; *n *= 6/experiment; two-way ANOVA with multiple comparisons; ns, not significant, *p < 0.05, ***p < 0.001, ****p < 0.0001; 6 independent experiments). MFI was normalized to controls.

We assessed CTFR-labeled T cells at six timepoints over five days to evaluate their activation kinetics *in vitro.* CD4^+^ and DP T cells proliferated faster, measured by the percentage of CTFR^LO^ cells, and produced more inflammatory cytokines (IFNγ and IL-17A) compared to CD8^+^ T cells. This difference became evident after 72 hours, with DP T cells proliferating significantly more than CD4^+^ T cells (**Figs 3A** and **3B**). By 96 and 120 hours, DP T cells continued to outpace both CD4^+^ and CD8^+^ T cells, while CD4^+^ T cells surpassed CD8^+^ T cells by Day 5.

Additionally, both CD4^+^ and DP T cells produced significantly higher levels of IFNγ and IL-17A compared to CD8^+^ T cells after 72 hours of stimulation, reinforcing the weak response of CD8^+^ T cells to RABV-G stimulation (**Figs 3C** and **3D**). Both CD4^+^ and DP T cells produced similar amounts of IFNγ over the course of five days compared to CD8^+^ T cells. DP T cells produced significantly more IL-17A than CD4^+^ and CD8^+^ T cells, with CD4^+^ T cells producing slightly more than CD8^+^ T cells. Overall, these findings indicate that both DP T cells and CD4^+^ T cells induce a rapid and robust IFNγ-dominated response upon RABV-G stimulation as early as 72 hours *in vitro*.

### The rabies vaccine induces a predominant T_H_1 immune response

**Fig 4:**
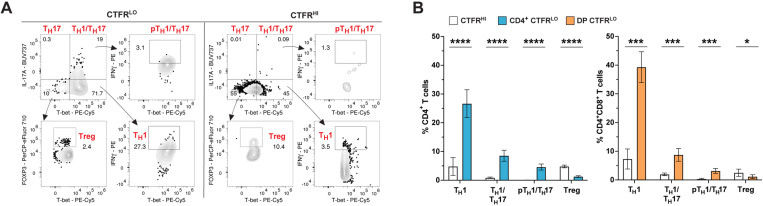
RABV-G-specific T cells are polarized T_H_1 cells. (A) Gating strategy and representative contour plots for lineage-defining subsets between CTFR^LO^ and CTFR^HI^ T cells to differentiate T_H_1 (T-bet^+^IFNγ^+^), T_H_17 (IL-17A^+^), T_H_1/T_H_17 (T-bet^+^IL-17A^+^), pathogenic T_H_1/T_H_17 (T-bet^+^IL-17A^+^IFNγ^+^), and Tregs (FOXP3^+^). (B) Quantification of (A) (mean ± SEM, *n* = 22 samples, paired two-tailed Wilcoxon test; *p < 0.05, ***p < 0.001, ****p < 0.0001; 4 independent experiments).

After demonstrating that CTFR^LO^ CD4^+^ and DP T cells respond to RABV-G stimulation by proliferating and producing IFNγ and IL-17A, we used flow cytometry to further characterize these cytokine-producing cells for expression of lineage-defining markers to define T_H_1, T_H_17, and Treg subsets (**Fig 4A**). Using validated markers for dog T cells [40–42], we found that both CTFR^LO^ CD4^+^ and DP T cells were predominantly T_H_1 (T-bet^+^IFNγ^+^) cells compared to their CTFR^HI^ counterparts (**Fig 4B**). Interestingly, a smaller fraction of CTFR^LO^ CD4^+^ and DP T cells also produced IL-17A, the vast majority of which were initially T-bet^–^ at Day 0 but had upregulated T-bet by Day 5 in response to RABV-G (S4A Fig). Therefore, we classified these cells as T_H_1/T_H_17 hybrid cells (T-bet^+^IL-17A^+^). A subset of these T_H_1/T_H_17 hybrid cells also produced IFNγ, defining them as pathogenic T_H_1/T_H_17 (pT_H_1/T_H_17) hybrid cells (T-bet^+^IL-17A^+^IFNγ^+^) [43]. A negligible percentage of T_H_17 cells lacking T-bet expression were observed at Day 5; these cells were excluded from downstream analysis (**Fig 4B**). To ensure that T-bet and IL-17A expression were not artificially upregulated by *in vitro* culture, we confirmed that mean fluorescence intensity (MFI) for T-bet and IL-17A remained low in CD8^+^ T cells, naïve cells, and CTFR^HI^ CD4^+^ and DP T cells compared to CTFR^LO^ CD4^+^ and DP T cells after five days of RABV-G stimulation (S5C Fig). Additionally, IL-17A production was specific to RABV-G-stimulation, as SEB stimulation induced T-bet expression without IL-17A production. Furthermore, SEB stimulation was non-specific, activating all subsets without preferential stimulation of any single subset (Figs S6C and S6D). These findings indicate that RABV-G-stimulated CTFR^LO^ cells exhibited a unique antigen-specific phenotype compared to the polyclonal activation seen in SEB-stimulated CTFR^LO^ cells. FOXP3 MFI was higher in CTFR^LO^ CD4^+^ and DP cells than in CTFR^HI^ cells, likely due to *in vitro* culture conditions given previous studies demonstrating that conventional CD4^+^ T cells non-specifically upregulate FOXP3 *in vitro* (S5C Fig) [44,45]. However, the majority of CD4^+^FOXP3^+^ Tregs were CTFR^HI^, demonstrating they did not proliferate when stimulated with RABV-G and therefore, likely play a minimal role in vaccine-induced responses.

Lastly, lineage subsets were evenly represented between the blood and the spleen, except for DP pT_H_1/T_H_17 cells, which were more abundant in the blood (S4B Fig). Taken together, our findings show that RABV-G stimulation induced proliferation of IFNγ-producing T_H_1 CD4^+^ and DP T cells, with minor subsets also demonstrating T_H_1/T_H_17 hybrid and pathogenic T_H_1/T_H_17 features. Most Treg cells did not proliferate, and the majority of lineage subsets were equally represented across both the blood and spleen.

### RABV-G-specific T cells exhibit a predominant T_EMRA_ phenotype

**Fig 5:**
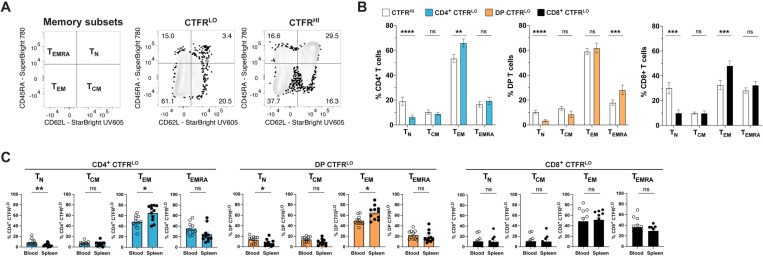
RABV-G stimulation induces a T_EMRA_ phenotype. (A) Gating strategy and representative contour plots to classify canine memory T cell subsets based on CD45RA and CD62L expression within CTFR^LO^ and CTFR^HI^ populations. (B) Quantification of (A) (mean ± SEM; *n* = 22 samples; paired two-tailed Wilcoxon test; ns, not significant, **p < 0.01, ***p < 0.001, ****p < 0.0001; 4 independent experiments). (C) Samples from (B) were analyzed by tissue type (blood vs. spleen) (mean ± SEM; n = 11/tissue type; two-tailed Mann-Whitney-U tests; *p < 0.05, **p < 0.01; 4 independent experiments).

We sought to further characterize RABV-G-stimulated T cells by their memory status. Canine memory T cells are defined by the markers CD45RA and CD62L and categorized into four groups: naïve T cells (T_N_); CD45RA^+^CD62L^+^, central memory (T_CM_); CD45RA^–^CD62L^+^, effector memory (T_EM_); CD45RA^–^CD62L^–^, and T_EMRA_; CD45RA^+^CD62L^–^ (**Fig 5A**) [46,47]. Based on this scheme, we found that both CTFR^LO^ CD4^+^ and CD8^+^ T cells were predominantly T_EM_ cells compared to CTFR^HI^ CD4^+^ and CD8^+^ T cells. While CTFR^LO^ DP T cells demonstrated a T_EMRA_ phenotype compared to their CTFR^HI^ counterparts. Therefore, re-expression of CD45RA in a terminally differentiated memory subset appears specific to CTFR^LO^ DP T cells (**Fig 5B**). This was further supported by comparing memory phenotypes between mock- and SEB-stimulated groups (Figs S6A and S6B). The lack of significance in the SEB-stimulated T_EMRA_ population (CTFR^HI^ vs CTFR^LO^) and the higher percentage of T_EM_ in both the SEB-stimulated CD4^+^ and DP CTFR^HI^ fractions emphasizes that polyclonal stimulation does not replicate RABV-G-induced phenotypes in CD4^+^ or DP T cells.

T_N_ cells were more abundant in the blood than in the spleen for both CTFR^LO^ CD4^+^ and DP T cells, whereas T_EM_ were more frequent in the spleen (**Fig 5C**). In contrast, no significant differences were observed in the distribution of CD8^+^ memory T cell subsets between the tissues. Overall, we showed that CTFR^LO^ CD4^+^ T cells were predominantly T_EM_ cells, while CTFR^LO^ DP cells demonstrated a T_EMRA_ phenotype.

### RABV-G specific T cell proliferation is not influenced by age or the time since the last rabies vaccination

**Fig 6:**
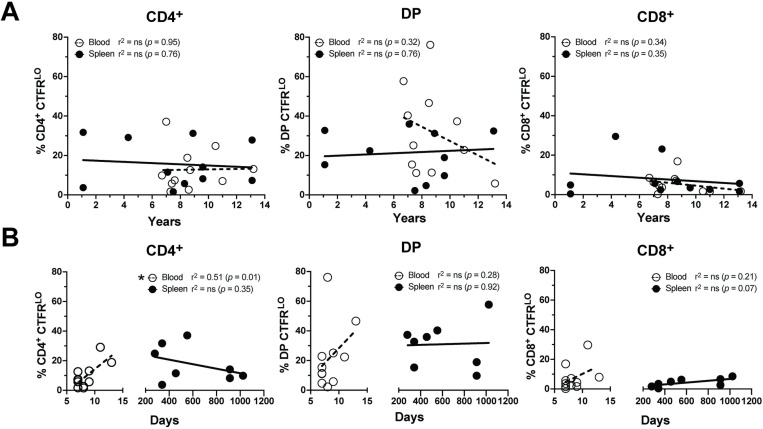
Re-stimulation of RABV-G-specific T cells is unbiased to age and the time elapsed since the last vaccination. (A) The frequency of RABV-G stimulated CTFR^LO^ T cells from each T cell subset were compared to the age of the dog by a simple linear regression model (*n *= 11/tissue type). (B) The frequency of RABV-G stimulated CTFR^LO^ T cells from either the blood or the spleen were compared to the number of days since the last rabies vaccination assessed by a simple linear regression model (*n *= 11/tissue type).

Immunosenescence is known to impact canine immunity, with blood-derived CD4^+^ and CD8^+^ T cells from dogs aged 8–10 years showing decreased proliferation compared to younger dogs in response to mitogen stimulation [46]. To assess potential age effects on RABV-G CTFR^LO^ populations, we used a linear regression model across T cell subsets (CD4^+^, DP, CD8^+^) and tissue types. No significant correlation between age and proliferative capacity was found (**Fig 6A**). We also investigated whether time since the last rabies vaccination affected T cell proliferation, hypothesizing that the proliferative ability of RABV-G-specific T cells would decrease over time in both the blood and spleen. Instead, we found a positive correlation between the days elapsed since vaccination and the *in vitro* proliferative capacity of CD4^+^ RABV-G CTFR^LO^ T cells in the blood as expected as recently activated effector T cells leave the lymphoid tissue and enter the blood. No significant differences were observed in the RABV-G CTFR^LO^ DP and CD8^+^ T cells from recently vaccinated blood samples or spleen samples collected over 200 days post-vaccination (**Fig 6B**). Three spleen samples had unknown vaccination statuses (S2 Table). These findings suggest that long-lived RABV-G-specific memory T cells are detectable in the spleen and that the peak of RABV-G-specific CD4^+^ T cell frequency may be beyond 7 days post-vaccination.

## Discussion

Nearly 90 million client-owned dogs live in the United States and must be vaccinated with a whole killed alum-adjuvanted rabies vaccine every 1–3 years [48,49], however, little is known about the adaptive immune responses dogs generate against these vaccines. The goal of this study was to identify and characterize canine rabies vaccine antigen-specific T cells after *in vitro* restimulation. From previous work studying rabies vaccine responses in humans and mice, the immunodominant response post-vaccine is generated against the rabies virus glycoprotein (RABV-G), which is the only surface-exposed protein on the rabies virus and required to facilitate viral entry into infected cells [18]. Few publications support the immunogenicity of other rabies virus proteins such as the nucleoprotein (RABV-N) [30,34]. As expected, we confirmed that RABV-G triggers a robust immune response in dogs, producing antibody titers that remain stable between vaccinations. Additionally, PBMCs stimulated with RABV-G generated a significantly higher IFNγ response, as measured by ELISpot, compared to RABV-N after 18–24 hours. Given the elevated IgG titers against RABV-G and the increased IFNγ production by bulk PBMCs, we hypothesized that a RABV-G-specific T cell population was being activated upon vaccination and could be detected upon restimulation. Previous studies support this hypothesis by demonstrating that virus-neutralizing antibodies (VNAs) specific to RABV-G protect dogs from a lethal rabies virus challenge [50]. In a separate study, dogs vaccinated with an inactivated rabies vaccine developed increased VNA titers specific to RABV- N that peaked at 3 weeks post-immunization [51]. This inactivated vaccine administered intramuscularly (IM) induced a higher percentage of FOXP3^+^CD25^+^ Tregs and a greater proportion of bulk CD3^+^CD4^+^ T cells compared to the control at 7 days post-immunization. Further research is needed to determine how the route of administration influences immune responses, particularly the increased presence of Tregs in dogs vaccinated IM versus to subcutaneously.

In humans and mice, peptide:MHC class II (pMHCII) tetramers are considered the gold standard to identify, track, and characterize antigen-specific CD4^+^ T cells [52–54]. In the absence of pMHCII tetramers for dogs and without an identified immunodominant RABV-G peptide, we sought to study antigen-specific T cells stimulated by a recombinant RABV-G protein. Using a flow cytometry panel with markers previously validated in dogs, we tracked antigen-specific T cell proliferation through CellTrace dye dilution [40,41,55]. We found that RABV-G-stimulated cells with diluted CellTrace Far Red (CTFR) dye, classified as CTFR^LO^ cells, exhibited distinct memory and lineage-defining markers compared to CTFR^HI^ cells, SEB-stimulated cells, and mock controls, indicating specificity for RABV-G. Proliferation assays showed robust responses from both CD4^+^ and DP T cells to RABV-G stimulation over five days, with proliferation detectable across various ages, vaccination histories, and tissue sources. This sustained response likely reflects a well-maintained memory T cell population, as dogs are required by law to be re-vaccinated against rabies every 1–3 years.

An *Ehrlichia chaffeensis* canine vaccine and infection model demonstrated similar findings where DP T cells were the dominant responding subset by proliferation assay, followed by CD4^+^ T cells [56]. While the role of DP T cells remains unclear across species, they are known to be multi-functional, acting as effector memory, cytotoxic, and IFNγ producing T cells in humans and non-human primates [57–59]. In healthy dogs, DP T cells make up about 2.4% of the total blood T cell population and can increase upon stimulation, though this was not observed when we stimulated PBMCs or splenocytes with RABV-G [42,56,60]. Canine DP T cells have been shown to be highly proliferative, produce IFNγ upon mitogen stimulation, express CD44 (indicating an effector phenotype), and are thought to arise from CD4^+^ T cell progenitors [42,55,61,62]. Interestingly, in pigs, CD8α serves as an activation marker for CD4^+^ T cells, indicative of an activated memory CD4^+^ T cell phenotype during pathogenic challenge [63,64]. Whether canine CD4^+^ T cells also upregulate CD8 upon activation remains unknown. In our study, RABV-G CTFR^LO^ DP T cells resembled CD4^+^ T cells because they produced similar amounts of IFNγ and IL-17A and when treated with an MHC class II blocking antibody, showed reduced proliferative capacity. Thus, canine DP T cells are likely highly activated CD4^+^ T cells that upregulate CD8 upon TCR engagement.

We observed that most RABV-G CTFR^LO^ CD4^+^ T cells were effector memory T cells (T_EM_), while CTFR^LO^ DP T cells exhibited a skewed terminally differentiated effector memory T cell phenotype that re-expressed CD45RA (T_EMRA_). In humans, CD4^+^ T_EMRA_ cells are seen in low frequencies in the blood (0.3–18%) compared to CD8^+^ T_EMRA_ cells (10–30%) [65–69], but are associated with protective immunity, notably in COVID-19 and dengue infections [66,70,71]. In healthy dogs, circulating CD4^+^ T_EMRA_ cells exist at frequencies more akin to human CD8^+^ T cells when analyzed directly *ex vivo* [47]. Similarly, in our study, CD4^+^, DP, and CD8^+^ T cells all contained comparable T_EMRA_ frequencies in the blood and the spleen (20–40% of total CD3^+^ T cells). Within the CTFR^LO^ populations, however, DP T cells predominantly displayed a T_EMRA_ phenotype, while CTFR^LO^ CD4^+^ T cells were largely T_EM_ cells, compared to their CTFR^HI^ counterparts. CTFR^LO^ CD8^+^ cells were no different in their CD62L/CD45RA expression compared to CD8^+^ CTFR^HI^ cells. Therefore, following rabies vaccination in dogs, CTFR^LO^ CD8^+^ seem to play a minimal role in the vaccine response.

By lineage-defining markers, CTFR^LO^ CD4^+^ and DP cells demonstrated a robust T_H_1 phenotype that expressed T-bet and produced IFNγ after five days of RABV-G stimulation *in vitro*. This correlated well with other canine vaccines that induce T_H_1 responses [3,72,73], human rabies vaccines [16,74], and other human vaccines that contain alum adjuvant (e.g., tetanus toxoid) [75]. In this study, a small population of CTFR^LO^ CD4^+^ and DP cells also produced IL-17A. Unexpectedly, nearly all IL-17A-producing cells were also T-bet^+^ after 5 days of antigenic restimulation. While T-bet is associated with a T_H_1-committed fate and IL-17A to a T_H_17 fate, their co-expression is characteristic of T_H_1/T_H_17 hybrid cells, which exhibit features of both [76,77]. T_H_1/T_H_17 hybrid cells are thought to arise under chronic inflammatory conditions [76,78]. Vaccines have been shown to generate potent T_H_1/T_H_17 hybrid cells in mice, non-human primates, and humans [79–82]. In dogs, similar cells have been documented in *Brucella canis* and *Ehrlichia chaffeensis* infections where CD4^+^ T cells produced both IFNγ and IL-17A simultaneously [56,83]. One limitation of this study is that an exhaustive panel of cytokines were not measured to characterize the T cell response to RABV-G stimulation over time. Future studies incorporating cytokines such as granzyme B, IL-2, TNF-α, and IL-4 might offer deeper insight into the heterogeneous T cell phenotypes involved. Nevertheless, it remains unclear whether CTFR^LO^ pathogenic T_H_1/T_H_17 cells represent a distinct subset or are part of the broader T_H_1/T_H_17 population within CD4^+^ and DP T cells. While a canine-specific RORγt antibody would have been valuable for identifying T_H_17-like cells, its lack of availability in dogs precluded more thorough validation. Therefore, further investigation is needed to determine whether CTFR^LO^ T_H_1/T_H_17 cells are specifically linked to rabies vaccination, induced by RABV-G stimulation *in vitro*, shaped by certain cell culture conditions, or are generated from chronic antigen exposure.

In summary, we characterized the specific T cell subsets responding to rabies vaccination after restimulation, which can provide insights into the efficacy of the vaccine and lay a foundation for phenotyping canine T cell subsets. We showed that the vaccine produces robust responses against the RABV-G from CD4^+^ and CD4^+^CD8^+^ double-positive T cells that produce IFNγ. We identified ways to phenotype canine T cells responding to an antigen based on memory status and lineage-defining characteristics. Continuation of this work will offer valuable insights into antigen-specific CD4^+^ T cell responses to other vaccines and can be broadly applied to immune-mediated conditions, where dogs can serve as an excellent naturally occurring model.

## Supporting information

S1 Fig B-ME is critical for the proliferation of canine T cells *in vitro.* Quantification of CTFR dilution in *n *= 7 spleen samples stimulated with either a mock negative control (0.1% DMSO), Concanavalin A, phorbol 12-myristate 13-acetate (PMA) and ionomycin, and Staphylococcal enterotoxin B (SEB) for five days in complete RPMI cell culture media containing β-mercaptoethanol (B-ME) or not. Significance was determined with a multiple paired two-tailed t-test.
(TIF)

S2 Fig Gating strategy for canine T cells. Representative contour plots showing the gating strategy to detect CD4^+^, CD8^+^, and DP T cell subsets in dogs based on gates on lymphocytes, singlets, CD3^+^Dump^–^ (CD14, CD21, CD11b, and dead cells). Then, based on CD8 and CD4 expression, T cells are further distinguished by CellTrace Far Red (CTFR) dilution by CTFR^HI^ and CTFR^LO^ gates.
(TIF)

S3 Fig RABV-G stimulation does not affect the overall percentage of T cell subsets after five days. **(A)** Representative contour plots of total CD3^+^ T cells by relative CD8 vs CD4 expression at a Day 0 direct *ex vivo*, Day 5 mock stimulated control, and Day 5 RABV-G-stimulated. **(B)** Quantification of (A) based on relative CD4, CD4/CD8, and CD8 expression within the three different groups (*n* = 22 samples; paired two-tailed Wilcoxon test).
(TIF)

S4 Fig Lineage-defining features change over five days but are equally represented in the blood and spleen. **(A**) Representative contour plots demonstrating IL-17A^+^ populations at Day 0 that acquire T-bet expression over the course of five days of RABV-G stimulation **(B)** Samples from Fig 4B were analyzed by tissue type (blood vs. spleen) (mean ± SEM; n = 11/tissue type; two-tailed Mann-Whitney-U tests; 4 independent experiments).
(TIF)

S5 Fig Biological controls validate a comprehensive canine T cell phenotyping panel. **(A)** Representative contour plots of splenocytes stimulated with RABV-G demonstrating how the CD4^+^ (blue), CD8^+^ (black), and DP (orange) T cell subsets are determined and subdivided into CTFR^LO^ and CTFR^HI^ populations based on proliferation dye dilution. **(B)** The populations defined in (A) were compared to an overall naïve population set on a broad gate including all three subsets (CD4^+^, CD8^+^, and DP), then gated on the naïve cells that express CD45RA and CD62L. **(C)** Each T cell subset was analyzed for their relative expression of lineage-defining markers (T-bet, IFNγ, IL-17A, FOXP3, CD62L, and CD45RA) were compared across the naïve, bulk CD8^+^, CD4^+^ CTFR^HI^, CD4^+^ CTFR^LO^, DP CTFR^HI^, and DP CTFR^LO^ populations.
(TIF)

S6 Fig Mock and SEB controls are distinctly different from RABV-G-stimulated T cells by memory and lineage phenotypes. **(A)** Representative contour plots of memory T cell subsets either stimulated with a mock negative control or a superantigen *Staphylococcus* enterotoxin B (SEB) in both CD4^+^ and DP T cell subsets. (**B**) Quantification of (A) (mean ± SEM; *n* = 22 samples; paired two-tailed Wilcoxon test; 4 independent experiments). (**C)** Representative contour plots of CD4^+^ and DP T-bet vs IL-17A expression in both mock (CTFR^HI^) and SEB (CTFR^LO^) stimulated conditions. (**D)** Quantification of (C) in either mock CTFR^HI^ CD4^+^ (left) or DP T cell (right) populations or SEB CTFR^HI^ and CTFR^LO^ CD4^+^ (left) or DP T cell (right) populations. (mean ± SEM; *n* = 22 samples; paired two-tailed Wilcoxon test; 4 independent experiments).
(TIF)

S7 Fig Recombinant RABV-N and Pasteur strain RABV-N are 99.3% similar. A BLOSUM62 Needleman-Wunsch global alignment of the Pasteur stain RABV-N (top sequence) and the LSBio RABV-N (bottom sequence). Amino acids that are not similar (amino acids unlikely to substitute for one another in nature) are shown in red, whereas amino acids that are similar (biochemically similar amino acids that could substitute for another without affecting protein function) are shown in blue.
(TIF)

S1 Table Proliferation assay spleen sample demographics. FS, female spayed. MN, male neutered. MI, male intact. IO, intraoperatively. PM, post-mortem.
(DOCX)

S2 Table Proliferation assay PBMC sample demographics. FS, female spayed. MN, male neutered. MI, male intact.
(DOCX)

S3 Table RABV antibody titer cohort demographics. FS, female spayed. MN, male neutered. MI, male intact.
(DOCX)

S4 Table Raw data and descriptive statistics for Figures 1–5. Raw data values for each dog (numbered 1 – X) shown for each figure, which are separated into distinct sheet tabs. Next to each dataset, descriptive statistics are provided including the 95% CI.
(XLSX)
