# Supplementary figures and images for: Rabies vaccination induces a CD4+ TEM and CD4+CD8+ TEMRA TH1 phenotype in dogs
Source: PLoS One. 2025 May 12;20(5):e0323823. doi: 10.1371/journal.pone.0323823 (PMC12068608; doi:10.1371/journal.pone.0323823)

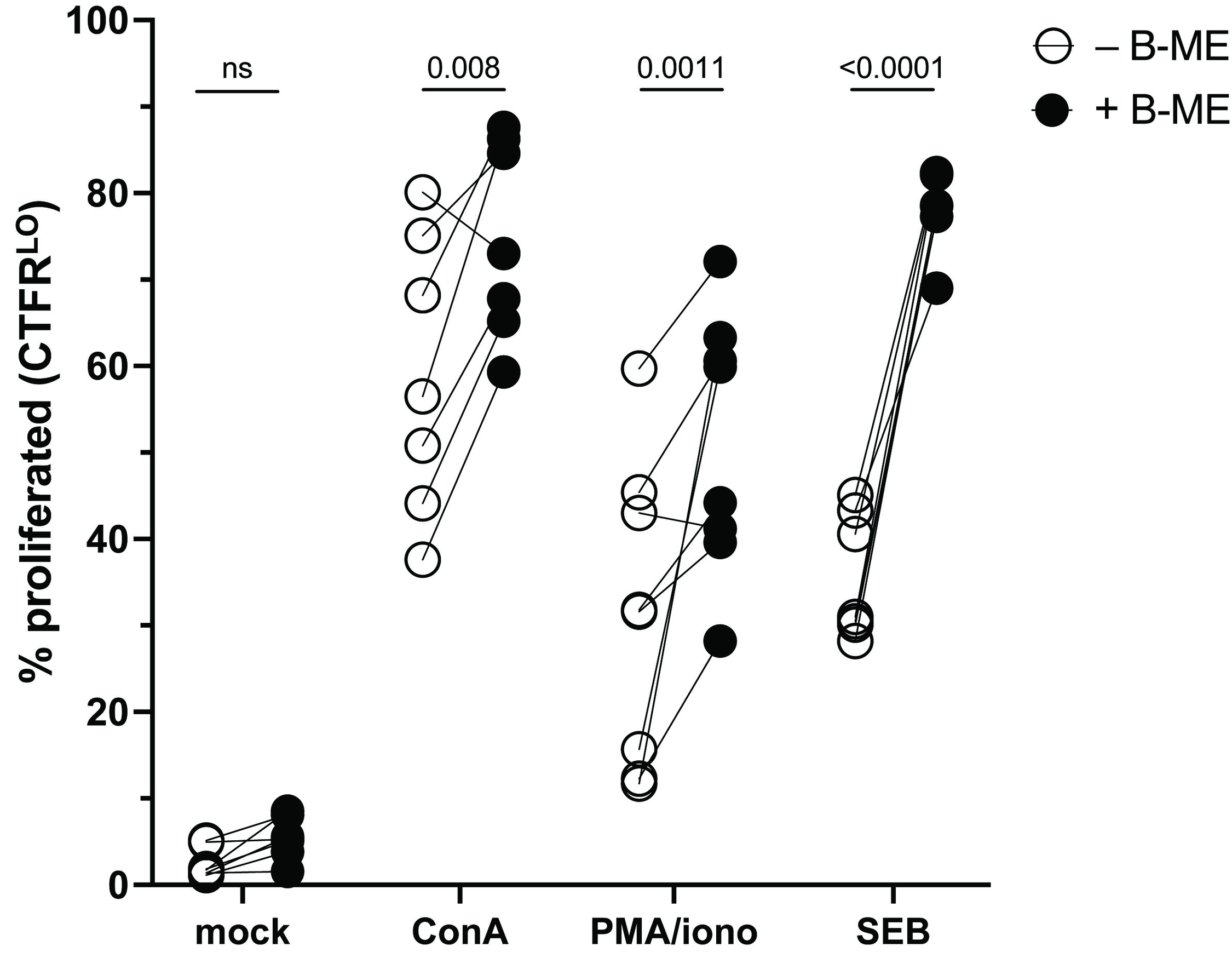

Supplement: S1 Fig — Quantification of CTFR dilution in n = 7 spleen samples stimulated with either a mock negative control (0.1% DMSO), Concanavalin A, phorbol 12-myristate 13-acetate (PMA) and ionomycin, and Staphylococcal enterotoxin B (SEB) for five days in complete RPMI cell culture media containing β-mercaptoethanol (B-ME) or not. Significance was determined with a multiple paired two-tailed t-test. (TIF) [file pone.0323823.s001.tif]

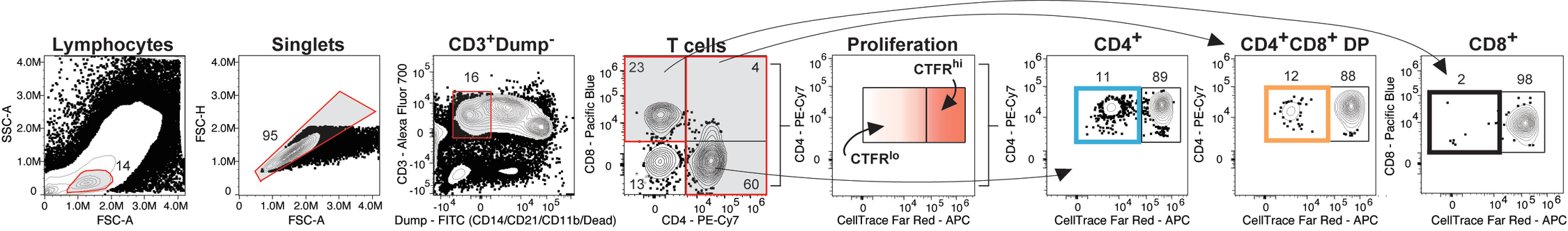

Supplement: S2 Fig — Representative contour plots showing the gating strategy to detect CD4+, CD8+, and DP T cell subsets in dogs based on gates on lymphocytes, singlets, CD3+Dump– (CD14, CD21, CD11b, and dead cells). Then, based on CD8 and CD4 expression, T cells are further distinguished by CellTrace Far Red (CTFR) dilution by CTFRHI and CTFRLO gates. (TIF) [file pone.0323823.s002.tif]

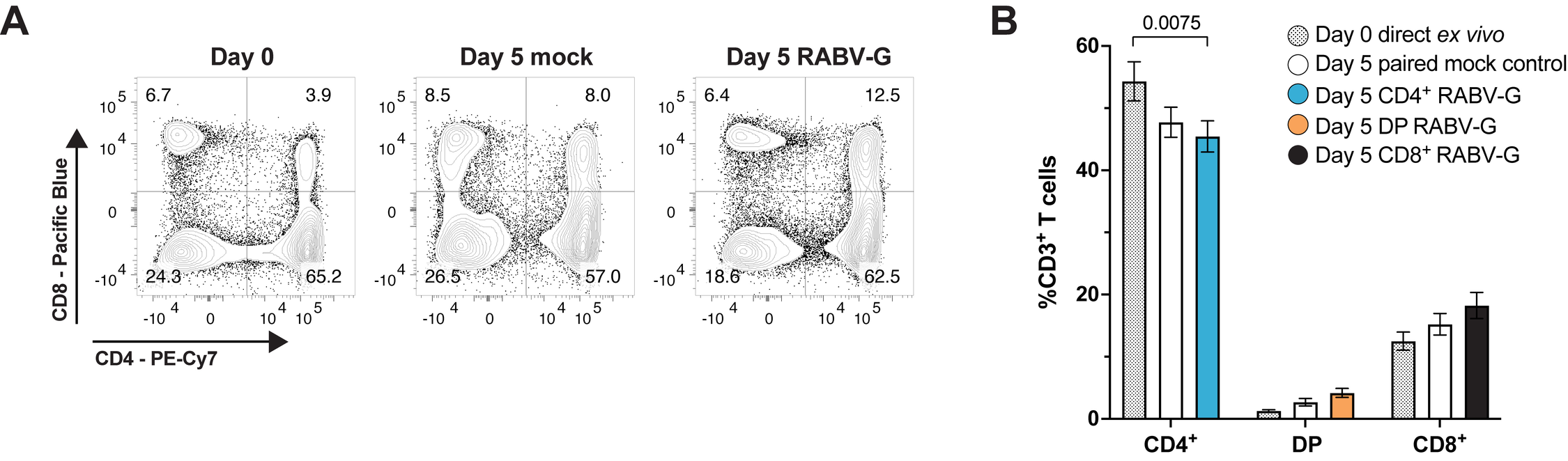

Supplement: S3 Fig — (A) Representative contour plots of total CD3+ T cells by relative CD8 vs CD4 expression at a Day 0 direct ex vivo, Day 5 mock stimulated control, and Day 5 RABV-G-stimulated. (B) Quantification of (A) based on relative CD4, CD4/CD8, and CD8 expression within the three different groups (n = 22 samples; paired two-tailed Wilcoxon test). (TIF) [file pone.0323823.s003.tif]

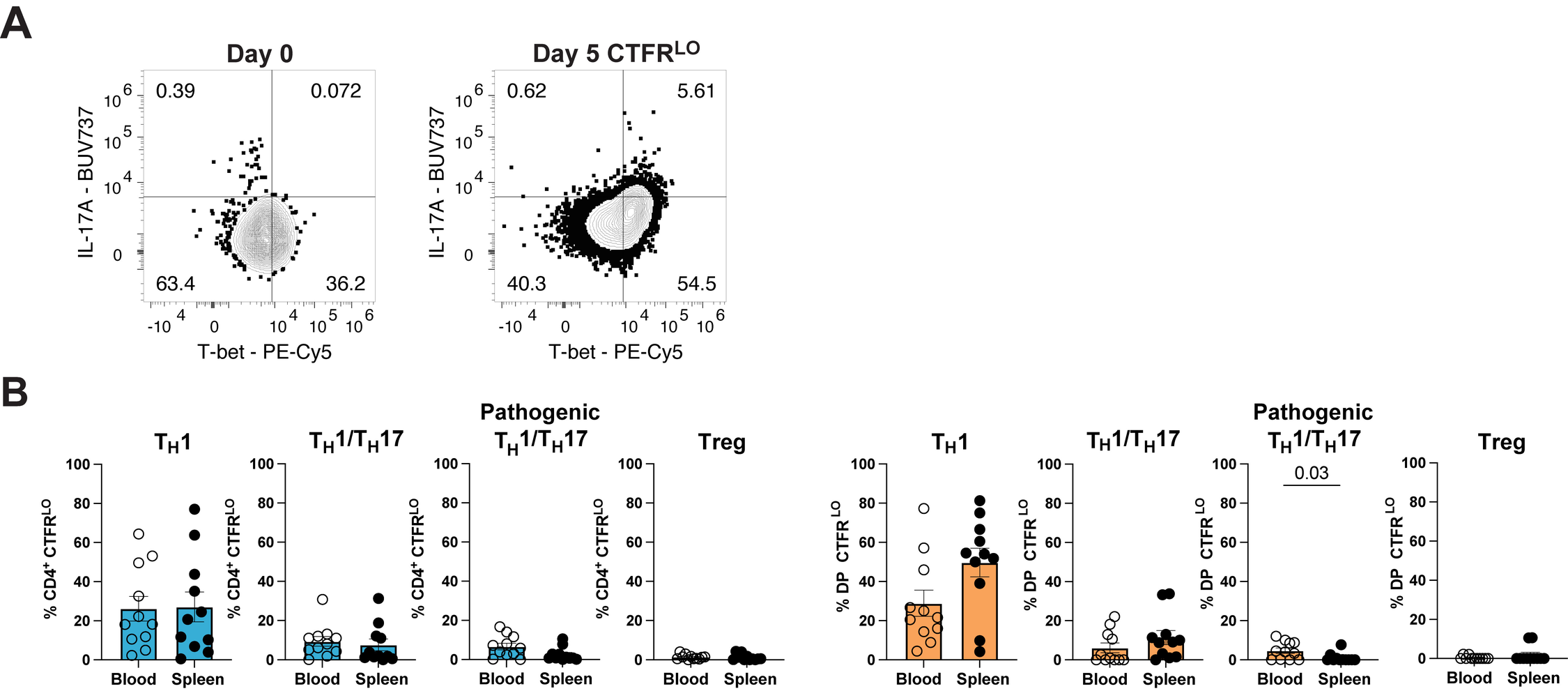

Supplement: S4 Fig — (A) Representative contour plots demonstrating IL-17A+ populations at Day 0 that acquire T-bet expression over the course of five days of RABV-G stimulation (B) Samples from Fig 4B were analyzed by tissue type (blood vs. spleen) (mean ± SEM; n = 11/tissue type; two-tailed Mann-Whitney-U tests; 4 independent experiments). (TIF) [file pone.0323823.s004.tif]

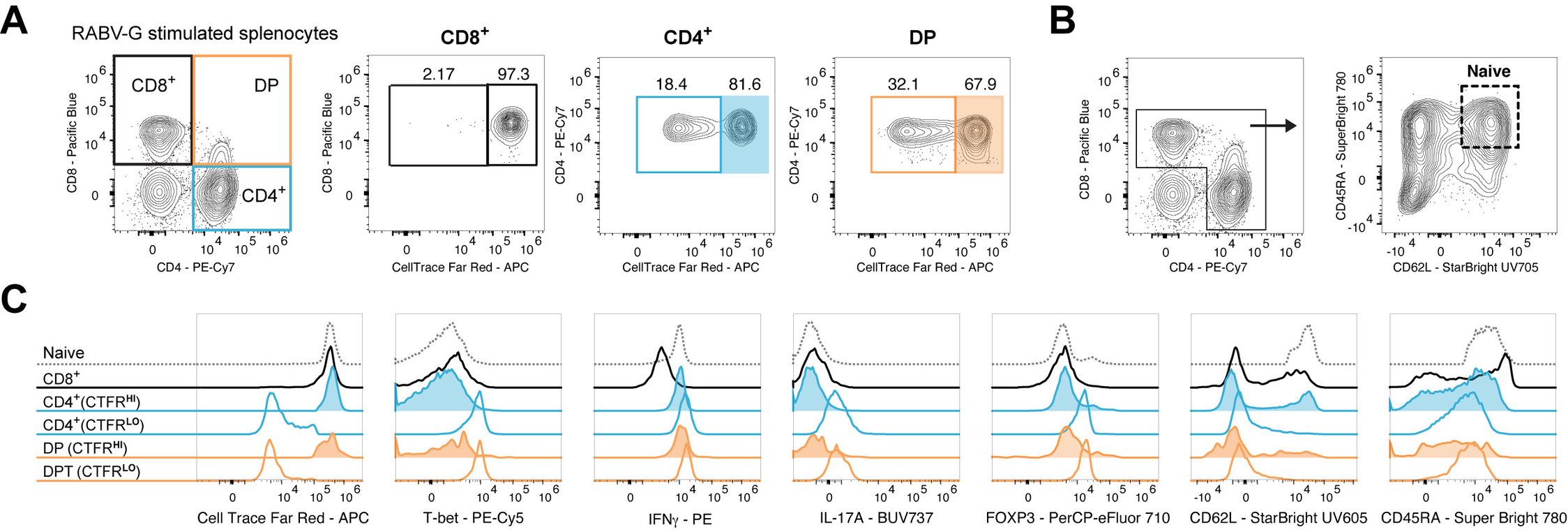

Supplement: S5 Fig — (A) Representative contour plots of splenocytes stimulated with RABV-G demonstrating how the CD4+ (blue), CD8+ (black), and DP (orange) T cell subsets are determined and subdivided into CTFRLO and CTFRHI populations based on proliferation dye dilution. (B) The populations defined in (A) were compared to an overall naïve population set on a broad gate including all three subsets (CD4+, CD8+, and DP), then gated on the naïve cells that express CD45RA and CD62L. (C) Each T cell subset was analyzed for their relative expression of lineage-defining markers (T-bet, IFNγ, IL-17A, FOXP3, CD62L, and CD45RA) were compared across the naïve, bulk CD8+, CD4+ CTFRHI, CD4+ CTFRLO, DP CTFRHI, and DP CTFRLO populations. (TIF) [file pone.0323823.s005.tif]

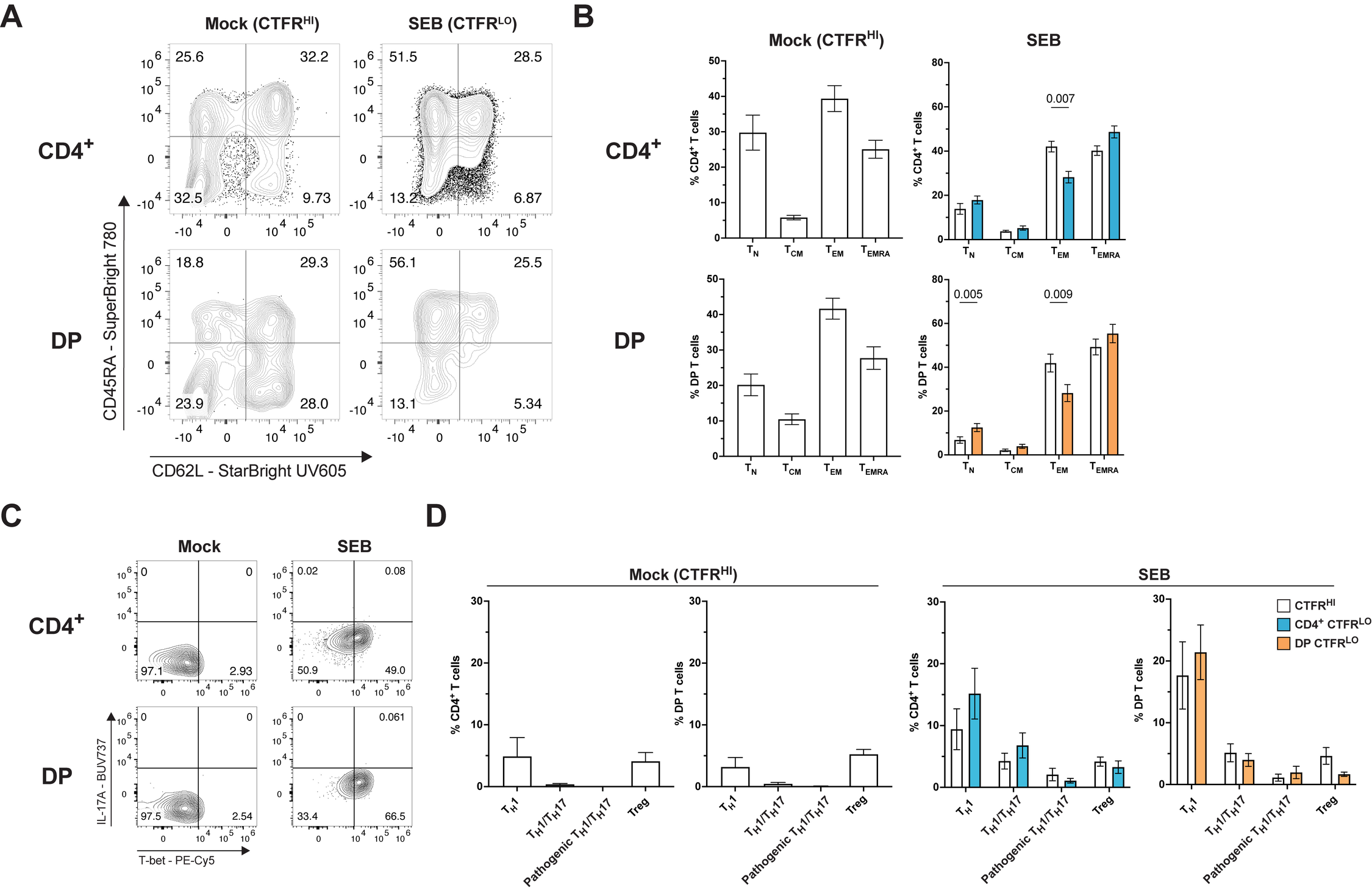

Supplement: S6 Fig — (A) Representative contour plots of memory T cell subsets either stimulated with a mock negative control or a superantigen Staphylococcus enterotoxin B (SEB) in both CD4+ and DP T cell subsets. (B) Quantification of (A) (mean ± SEM; n = 22 samples; paired two-tailed Wilcoxon test; 4 independent experiments). (C) Representative contour plots of CD4+ and DP T-bet vs IL-17A expression in both mock (CTFRHI) and SEB (CTFRLO) stimulated conditions. (D) Quantification of (C) in either mock CTFRHI CD4+ (left) or DP T cell (right) populations or SEB CTFRHI and CTFRLO CD4+ (left) or DP T cell (right) populations. (mean ± SEM; n = 22 samples; paired two-tailed Wilcoxon test; 4 independent experiments). (TIF) [file pone.0323823.s006.tif]

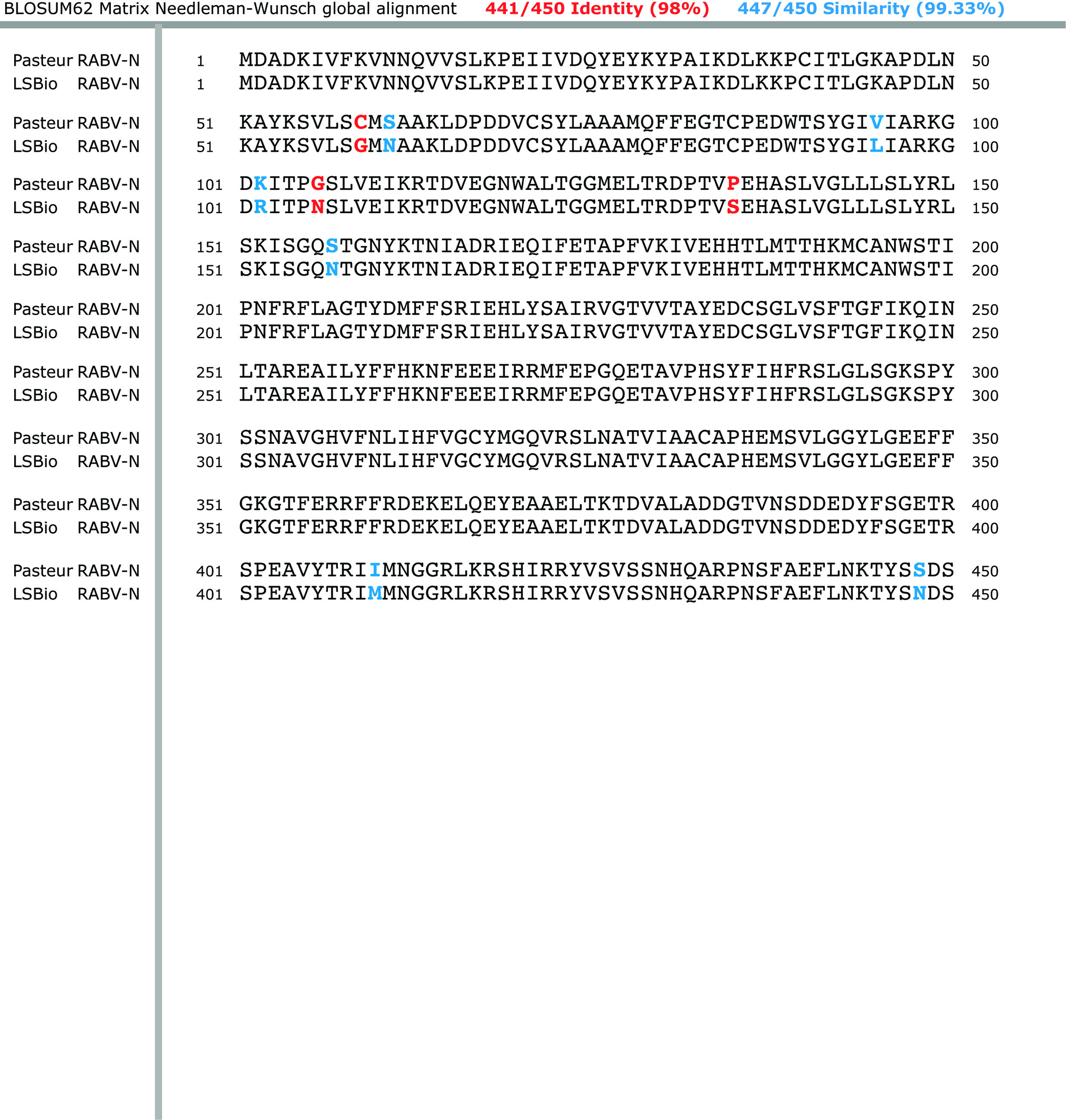

Supplement: S7 Fig — A BLOSUM62 Needleman-Wunsch global alignment of the Pasteur stain RABV-N (top sequence) and the LSBio RABV-N (bottom sequence). Amino acids that are not similar (amino acids unlikely to substitute for one another in nature) are shown in red, whereas amino acids that are similar (biochemically similar amino acids that could substitute for another without affecting protein function) are shown in blue. (TIF) [file pone.0323823.s007.tif]
