# Supplementary material for: Rabies vaccination induces a CD4+ TEM and CD4+CD8+ TEMRA TH1 phenotype in dogs
Source: PLoS One. 2025 May 12;20(5):e0323823. doi: 10.1371/journal.pone.0323823 (PMC12068608; doi:10.1371/journal.pone.0323823)
Supplement: S1 Table — FS, female spayed. MN, male neutered. MI, male intact. IO, intraoperatively. PM, post-mortem. (DOCX) [file pone.0323823.s008.docx]

| **ID** | **Age (years)** | **Sex** | **Breed** | **Time since last vaccine (days)** | **Collection type** |
| --- | --- | --- | --- | --- | --- |
| 1 | 9.6 | MN | Mixed Breed | 913 | IO |
| 2 | 1.1 | MI | Hound | 341 | PM |
| 3 | 1.1 | MI | Hound | 341 | PM |
| 4 | 7.1 | FS | Golden Retriever | 456 | IO |
| 5 | 13.1 | FS | Cocker Spaniel | Unknown | IO |
| 6 | 13.1 | FS | Cocker Spaniel | Unknown | IO |
| 7 | 8.9 | FS | Rottweiler | Unknown | IO |
| 8 | 9.6 | MN | Mixed Breed | 913 | IO |
| 9 | 7 | MN | German Shepherd | 553 | PM |
| 10 | 6.7 | MN | Golden Retriever | 1024 | IO |
| 11 | 10.5 | FS | Labrador Retriever | 279 | IO |

**S1 Table. Proliferation assay spleen sample demographics.** FS, female spayed. MN, male neutered. MI, male intact. IO, intraoperatively. PM, post-mortem.
