# Supplementary material for: Rabies vaccination induces a CD4+ TEM and CD4+CD8+ TEMRA TH1 phenotype in dogs
Source: PLoS One. 2025 May 12;20(5):e0323823. doi: 10.1371/journal.pone.0323823 (PMC12068608; doi:10.1371/journal.pone.0323823)
Supplement: S2 Table — FS, female spayed. MN, male neutered. MI, male intact. (DOCX) [file pone.0323823.s009.docx]

| **ID** | **Age (years)** | **Sex** | **Breed** | **Time since last vaccine (days)** |
| --- | --- | --- | --- | --- |
| 1 | 8.7 | MN | Standard Poodle | 7 |
| 2 | 7.6 | FS | Standard Poodle | 7 |
| 3 | 8.6 | MN | Standard Poodle | 7 |
| 4 | 8.3 | MN | Labradoodle | 7 |
| 5 | 4.3 | MN | Labrador Retriever | 11 |
| 6 | 8.5 | MN | Mixed Breed | 13 |
| 7 | 7.5 | FS | Basset Hound | 8 |
| 8 | 8.6 | MN | Weimaraner | 8 |
| 9 | 7.3 | MI | Deerhound Scottish | 7 |
| 10 | 7.4 | FS | Mixed Breed | 9 |
| 11 | 13.2 | MN | Labrador Retriever | 9 |

**S2 Table. Proliferation assay PBMC sample demographics.** FS, female spayed. MN, male neutered. MI, male intact.
