# Supplementary material for: Rabies vaccination induces a CD4+ TEM and CD4+CD8+ TEMRA TH1 phenotype in dogs
Source: PLoS One. 2025 May 12;20(5):e0323823. doi: 10.1371/journal.pone.0323823 (PMC12068608; doi:10.1371/journal.pone.0323823)
Supplement: S3 Table — FS, female spayed. MN, male neutered. MI, male intact. (DOCX) [file pone.0323823.s010.docx]

| **ID** | **Age (years)** | **Sex** | **Breed** |
| --- | --- | --- | --- |
| 1 | 4.5 | FS | Labrador Retriever |
| 2 | 7.6 | MN | Basset Hound |
| 3 | 4.3 | MI | Standard Poodle |
| 4 | 8.5 | MI | Mixed Breed |
| 5 | 8.3 | FS | Golden Retriever |
| 6 | 5.6 | MI | Doberman Pinscher |
| 7 | 8.1 | FS | Labrador Retriever |
| 8 | 4.3 | MN | Weimaraner |
| 9 | 7.7 | MN | Standard Poodle |
| 10 | 2.3 | FS | Mixed Breed |
| 11 | 0.3 | MI | American Staffordshire Terrier |

**S3 Table. RABV antibody titer cohort demographics.** FS, female spayed. MN, male neutered. MI, male intact.
